# Anti-HMGCR positive immune-mediated necrotizing myopathy with associated neutrophilic urticarial dermatosis: A case report

**DOI:** 10.1016/j.jdcr.2024.08.049

**Published:** 2024-10-15

**Authors:** Kathleen M. Fletcher, Nasro A. Isaq, Julia S. Lehman, Nessa Aghazadeh Mohandesi

**Affiliations:** a Department of Dermatology, Mayo Clinic, Rochester, Minnesota; b Department of Laboratory Medicine & Pathology, Mayo Clinic, Rochester, Minnesota

**Keywords:** immune mediated necrotizing myopathy, neutrophilic urticarial dermatosis

## Introduction

Anti-3-hydroxy-3-methylglutaryl-coenzyme A reductase (HMGCR) positive immune-mediated necrotizing myopathy (IMNM) is a rare subtype of idiopathic inflammatory myopathy potentially associated with statin exposure.^1^ It is characterized by significant myalgia and symmetrical proximal muscle weakness with elevated serum creatine kinase and necrotic muscle fibers on muscle biopsy.^1^ Historically, anti-HMGCR positive IMNM has been regarded to be a muscle-limited disease without cutaneous involvement. However, in recent years there have been few reports of varying cutaneous manifestations, the majority of which describe a dermatomyositis-like cutaneous eruption with skin biopsies revealing perivascular lymphocytic infiltrates, vacuolization of the basal layer, and increased dermal mucin consistent with their clinical presentation.^2^, ^3^, ^4^ Other described cutaneous manifestations include nonspecific exanthema, plaques, or hair loss.^5^ Herein, we report the first case of anti-HMGCR positive IMNM associated with neutrophilic urticarial dermatosis confirmed on dermatopathology.

## Case report

A previously healthy 36-year-old African American woman presented for evaluation of a painful and burning rash involving her bilateral malar cheeks, chin, and chest for about 8 months duration. A few months prior to the development of this rash, she was noted to have elevated liver enzymes of unknown etiology. She then progressed to develop severe proximal muscle weakness hindering her ability to perform activities of daily life prompting further investigation.

**Fig 1:**
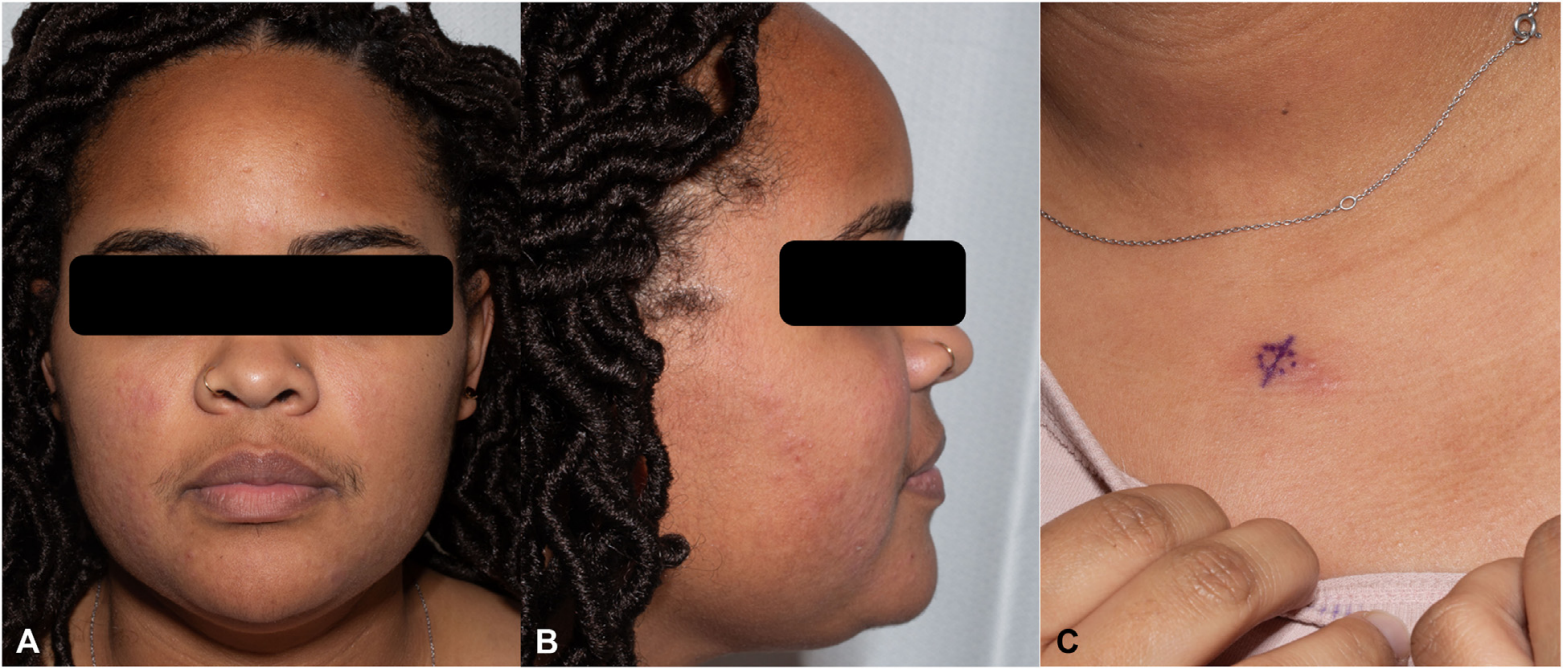
Erythematous papules involving the bilateral malar cheeks extending along the jawline to the chin (**A** and **B**), as well as erythematous papules coalescing into a plaque on the anterior chest (**C**).

**Fig 2:**
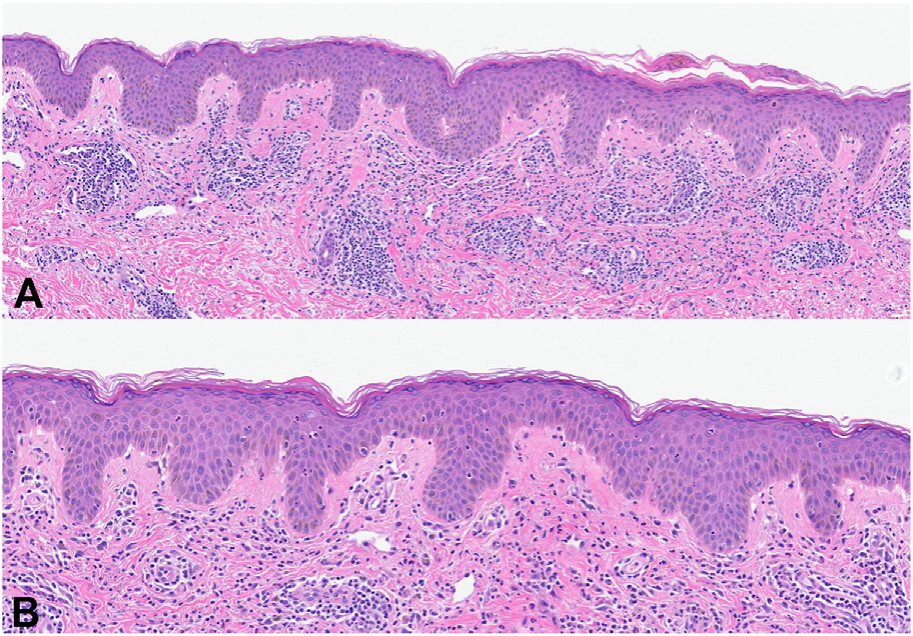
Neutrophilic epitheliotropism, as well as perivascular and interstitial neutrophils with karyorrhectic debris, is seen. **A,** Hematoxylin and eosin stain; 20× digital zoom; **B,** Hematoxylin and eosin stain; 32× digital zoom.

Physical examination revealed flesh-colored to erythematous papules involving the bilateral malar cheeks extending along the jawline to the chin, as well as erythematous papules coalescing into a plaque in the center of her chest (Fig 1). She denied any associated photosensitivity, recent infections, changes in medications, or vaccinations. A punch biopsy was obtained from the chest which revealed perivascular and interstitial neutrophilic inflammation with neutrophilic epitheliotropism, karyorrhexis, and dermal edema, consistent with neutrophilic urticarial dermatosis (Fig 2). Accompanying direct immunofluorescence was negative.

Laboratory evaluation revealed significantly elevated creatine kinase to 11, 575 U/L (26-192 U/L), lactate dehydrogenase, aldolase, and liver function tests. Serum anti-HMGCR antibody levels were elevated to 474 CU (normal <20.0 CU). Anti-signal recognition particle autoantibodies, myomarker panel, monoclonal gammopathy screen, hepatitis serology, HIV, antinuclear antibody, and extractable nuclear antigens were negative. Whole-body positron emission tomography demonstrated patchy fluorodeoxyglucose uptake in the upper and lower extremity proximal muscles consistent with myositis. Electromyography showed evidence of a severe diffuse myopathy with frequent fibrillation, indicating the presence of muscle necrosis. Together, this was consistent with the diagnosis of anti-HMGCR positive IMNM. Muscle biopsy was deferred as there was sufficient evidence to support the diagnosis and to prevent further delay of treatment initiation.

She was started on solumedrol, intravenous immunoglobulin, and methotrexate for management. On this regimen, her proximal muscle weakness, inflammatory muscle markers, and cutaneous eruption improved rapidly over the next 3 months. The neutrophilic urticarial dermatosis completely resolved after 3 infusions of intravenous immunoglobulin.

## Discussion

Inflammatory myopathies are a heterogeneous group of autoimmune diseases characterized by muscle weakness, elevated serum muscle enzymes, autoantibodies, and inflammation noted on muscle biopsy. This group of disorders includes dermatomyositis, inclusion-body myositis, anti-synthetase syndrome, and immune-mediated necrotizing myopathy. Diagnosis requires careful clinical history, physical examination, review of systems, serum muscle enzyme levels, electromyography, muscle biopsy, and review of serologic autoantibodies. These elements, particularly the myositis-specific autoantibodies, are evaluated together to determine the subtype of inflammatory myopathies.^6^^,^^7^

Anti-HMGCR positive IMNM is a rare subtype of idiopathic inflammatory myopathy that seldom presents with extramuscular involvement.^1^ As HMGCR is the pharmacologic target of statins, this entity is frequently associated with a history of statin exposure. However, it is estimated that about one-third of patients have no prior statin exposure, similar to the patient reported in this case.^7^ Additionally, some studies suggest that there may be an association with an underlying malignancy and age-appropriate cancer screening is recommended. The patient in this report had no identifiable malignancy in association with the development of her disease.^1^

Prior reports have described a dermatomyositis-like cutaneous eruption in association with anti-HMGCR positive IMNM.^2^, ^3^, ^4^ In these cases, patients have presented with erythematous-to-violaceous photodistributed papules and plaques involving the anterior chest, upper back, proximal upper and lower extremities, dorsal hands, and periocular regions. However, only a few of these prior studies have accompanying histopathology from the skin confirming this diagnosis.^2^ The presented case is unique as it demonstrates anti-HMGCR positive IMNM presenting with biopsy-proven neutrophilic urticarial dermatosis, highlighting the importance of histology in establishing this diagnosis. The distinct clinical differences between anti-HMGCR positive IMNM and dermatomyositis are outlined in Table I.

**Table I:** Immune-mediated necrotizing myopathy versus dermatomyositis

	Myositis-specific autoantibodies	Proximal muscle weakness	Creatine kinase (CK)	Muscle biopsy findings	Clinical associations
Immune-mediated necrotizing myopathy (IMNM)					
Anti-signal recognition particle (anti-SRP)	Anti-SRP	+	Elevated	Muscle fiber necrosis (not required for diagnosis)	• More severe muscle involvement • Common extra-muscular features such as cardiac involvement, interstitial lung disease, and dysphagia • May respond best to regimens that include rituximab
Anti-3-hydroxy-3-methylglutaryl-coA reductase (anti-HMGCR)	Anti-HMGCR	+	Elevated	Muscle fiber necrosis (not required for diagnosis)	• Association with statin exposure (approximately two-thirds of cases) • Typically isolated skeletal muscle weakness • Responds well to IVIG
Seronegative	None	+	Elevated	Muscle fiber necrosis (required for diagnosis)	• Often associated with underlying malignancy • Less understood than seropositive IMNM
Dermatomyositis	• Anti-Mi-2∗ • Antinuclear matrix protein 2 [NXP2]∗ • Anti–transcription intermediary factor 1 [TIF1]∗ • Anti–melanoma differentiation-associated protein 5 [MDA5]∗	+/− (amyopathic subtypes exist)	Elevated or normal	Perifascicular atrophy (not required for diagnosis)	• Characteristic cutaneous involvement (including but not limited to): ○ Heliotrope rash ○ V sign ○ Shawl sign ○ Holster sign ○ Mechanic hands ○ Gottron papules

*IVIG*, Intravenous immunoglobulin.

∗ Each dermatomyositis-specific autoantibody corresponds to a unique clinical phenotype not described here.

Neutrophilic urticarial dermatosis is a rare dermatosis associated with systemic autoimmune and autoinflammatory diseases including lupus erythematosus, adult-onset Still’s disease, or Schnitzler syndrome.^8^ It is characterized by perivascular and interstitial neutrophilic infiltrate with accompanying leukocytoclasia, but without vasculitis or significant dermal edema on histopathology.^8^

Further studies detailing cases of anti-HMGCR positive IMNM and the various cutaneous manifestations of this disease are needed to further elucidate the prevalence and morphology of its cutaneous involvement. Furthermore, this case report demonstrates the importance of dermatologists being able to recognize IMNM as another potential inflammatory myopathy, how to distinguish it from similar conditions, such as dermatomyositis, and its association with neutrophilic urticarial dermatosis.

## Conflicts of interest

None disclosed.
